# Supplementary material for: A Global Screen for Assembly State Changes of the Mitotic Proteome by SEC-SWATH-MS
Source: Cell Syst. 2020 Feb 26;10(2):133–155.e6. doi: 10.1016/j.cels.2020.01.001 (PMC7042714; doi:10.1016/j.cels.2020.01.001)

Q9Y3A3 | PHOCN_HUMAN | MOB4 MOB3 MOBKL3 PHOCN PREI3 CGI−95
Monomer MW [kDa]: 26.032 Monomer expected elution fraction: 52

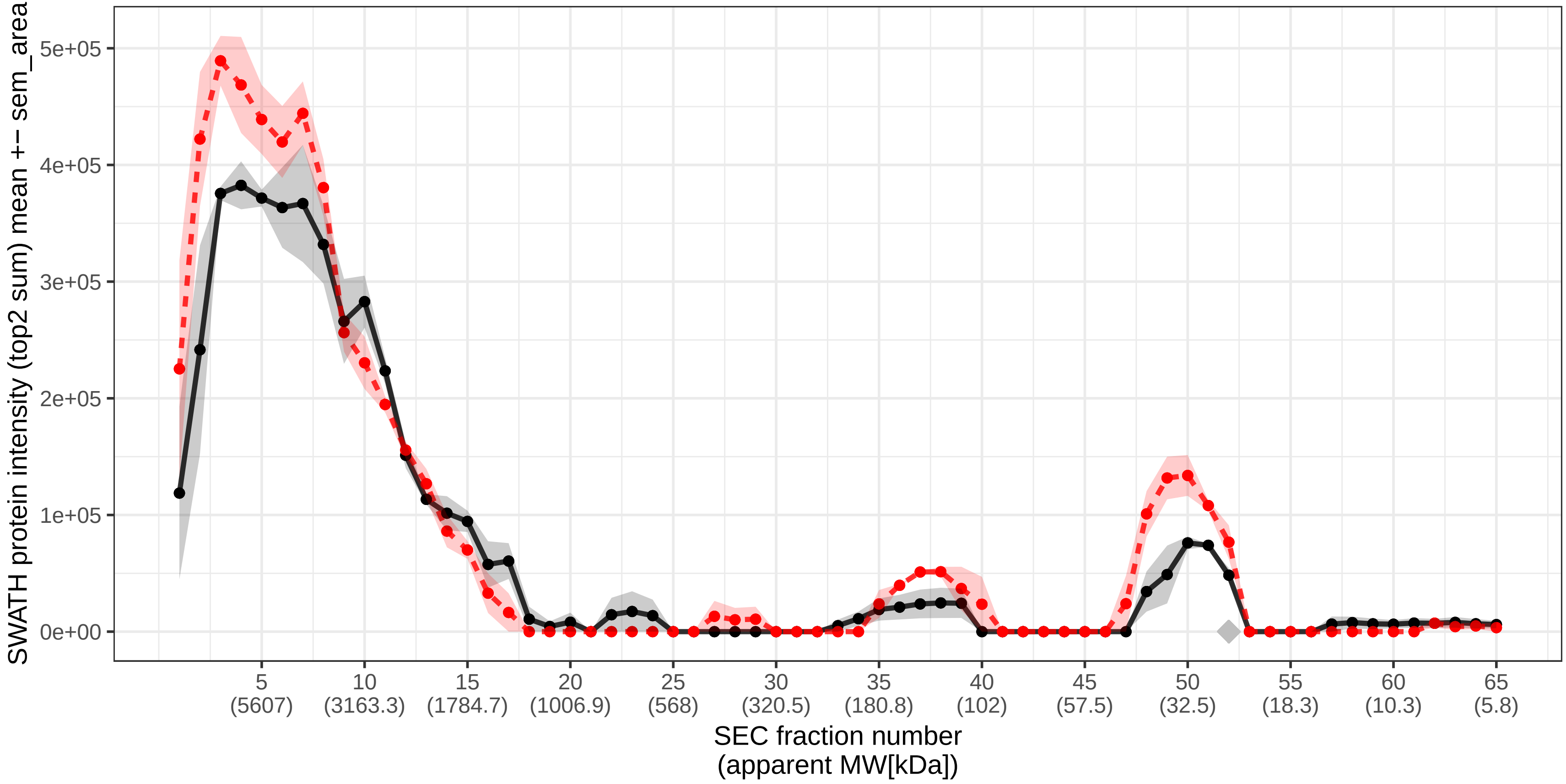

Supplement: Data S1. SEC-SWATH-MS Protein Chromatograms, Related to Figure 1 [file mmc6.zip › SECchrom_Q9Y3A3_PHOCN_HUMAN_MOB4_MOB3_MOBKL3_PHOCN_PREI3_CG.pdf]
